# Biomarkers of long COVID in children and young adults: a scoping review

**DOI:** 10.1007/s00431-026-06789-7

**Published:** 2026-02-16

**Authors:** Bettina Camara, Danilo Buonsenso

**Affiliations:** 1 Faculty of Medicine and Surgery, Università Cattolica del Sacro Cuore, Rome, Italy; 2 Department of Woman and Child Health and Public Health, Fondazione Policlinico Universitario A. Gemelli IRCCS, Largo A. Gemelli 8, 00168 Rome, Italy; 3 Area Pediatrica, Dipartimento Di Scienze Della Vita E Sanità Pubblica, Università Cattolica del Sacro Cuore, Rome, Italy

**Keywords:** Long COVID, Pediatrics, SARS-CoV-2, Biomarkers

## Abstract

Following the SARS-CoV-2 pandemic, a significant percentage of people are now experiencing long-term symptoms, despite a continuing lack of concrete documentation of physiological and risk profiles that hinders diagnosis and treatment, particularly in pediatric contexts. This review aims to highlight the existing evidence for measurable physiological markers for post-acute sequelae of SARS-CoV-2 infection (long COVID) in children, adolescents, and young adults. Titles providing data related to measurable biomarkers distinguishing young long COVID patients from controls were compiled and analyzed. Results were displayed in table and diagram form for optimal qualitative evaluation of the relationship between markers and symptomatology within the context of each organ system. Only human studies published in English, Italian, Portuguese, German, and Spanish between the 5th of February 2025 and the 31st of December 2025 were considered, and no other time constraints were applied. Following search and criteria evaluation, nine studies were included, totaling 41 occurrences identified in diseased patients with statistically significant variation from healthy controls. Markers suggest the presence of organic manifestations based on published literature, although more data and future studies will be necessary to establish clear connections.

*Conclusion*: The data compiled for this review adds to the body of evidence indicating a physiological manifestation of long COVID and its consequences. Further investigation into potential risk factors, pre- and post-pubescent manifestations, and specific inflammatory and immune pathways will be necessary for a more concrete understanding of long COVID and its effects on children, adolescents, and young adults.

**Table:** 

**What is Known:** • *Long COVID is estimated to affect a significant population of patients, despite the lack of concrete physiological diagnostic and prognostic measures*. • *Pediatric incidence of the disease is still largely debated, and published data are scarce*.	
**What is New:** • *A total of 41 biomarker occurrences were identified by selected studies, which were consistent with expected physiology behind reported symptoms*. • *The body of data discussed suggests the presence of physiological phenomena behind the long-term symptoms experienced by pediatric long- COVID patients*.

**Supplementary Information:**

The online version contains supplementary material available at 10.1007/s00431-026-06789-7.

## Introduction

Global spikes in SARS-CoV-2 infections have resulted in an unprecedented number of cases of individuals reporting long-term symptomatology beyond the expected period of recovery. Patients experiencing post-acute sequelae of SARS-CoV-2 infection (PASC), also known as “long COVID,” can experience a wide range of symptoms with the involvement of varied organ systems. The World Health Organization has suggested that symptoms persisting past 3 months, impairing daily functioning, are to be considered long COVID [1]; however, diagnostic characterization has been challenging due to the range of systems affected and the lack of specific biomarkers identified. Chiefly challenging, pediatric cases present particular hurdles due to the heterogeneity of cases and symptoms when compared to adults, as well as clinical study limitations.

Considering the 777 million reported cases of SARS-CoV-2 infection as of March 2025 [2], it is expected that approximately 10% of patients will develop into long COVID based on past reported rates [3]. Pediatric incidence within this estimated population is, however, highly debated [4, 5]. Symptom manifestation and management can pose a significant obstacle for patients and healthcare providers, and understanding the mechanisms of disease behind long COVID has been an ongoing effort for the scientific community. Initially overshadowed by acute respiratory symptoms and cardiovascular complications, long COVID symptoms in children, adolescents, and young adults tend to last several months [6], and accompany the onset of secondary conditions such as diabetes mellitus [7], autoimmunity [8], respiratory insufficiency [9], renal dysfunction [10] and others. Moreover, pediatric long COVID cases exhibit parallels with other viral-onset medical conditions, such as myalgic encephalomyelitis/chronic fatigue syndrome (ME/CFS) [11] and postural orthostatic tachycardia syndrome (POTS) [12]. These similarities have spurred interdisciplinary research efforts aimed at unraveling the complex pathogenetic mechanisms underlying long COVID, paving the way for a more comprehensive understanding of the syndrome and its age-specific clinical manifestation.

This scoping review aims to provide an updated look specifically into the available evidence of systemic biomarkers in pediatric patients and cohorts suffering from long COVID. Recent years have seen an increase in controlled studies aiming to analyze the existence of physiological biomarkers that could serve as indicators of disease. The compilation of new evidence will add to previously published works [13], and provide an outlook into the possibility of greater future diagnostic and prognostic accuracy for patients, particularly children, adolescents, and young adults.

## Methodology

This scoping review was conducted according to the checklist provided by the Preferred Reporting Items for Systematic reviews and Meta-Analyses extension for Scoping Review (PRISMA-ScR) [14]. The Joanna Briggs Methodology for scoping reviews was used as a guide for the development of this review protocol, taking the 2020 updates into consideration [15]. Registration of this protocol can be found at Open Science Framework (10.17605/OSF.IO/TNGYW), which was followed without deviations [16].

This scoping review was based on a priori research framework, specifically the population–concept–context (PCC) approach. The population of interest comprised pediatric patients with a documented history of acute SARS-CoV-2 infection and a subsequent diagnosis of long COVID. The central concept of the review was the identification and characterization of reported biological biomarkers associated with pediatric long COVID. The context included human retrospective and prospective clinical studies conducted in post-acute and follow-up care settings, without restrictions on geographic location or publication date. This framework informed the eligibility criteria, search strategy, data extraction, and charting of results.

## Eligibility criteria

Retrospective or prospective studies including pediatric patients and young adults (0–25 years) with a previous diagnosis of acute SARS-CoV-2 infection and sequential diagnosis of long COVID were selected. Eligible studies were chosen based on reported biomarkers in PACS/long COVID/post COVID condition patients, such that gray literature, qualitative studies, and reports were not included.

The patient population was not excluded based on sex or previous health conditions. Given significant gaps in our understanding of long COVID, particularly under the pediatric context, the definitions of long COVID were not subjected to scrutiny as long as the diagnosis process was performed by qualified professionals. Rather, the methodology and specificity of the biomarkers were considered, based on patients’ post-COVID inflammatory responses, to allow for the evaluation of valid future biomarker candidates. A broader scope of patients allowed for demographic analysis of PACS incidence in the pediatric population and added to the growing body of analytical data. Only human studies have been considered. Clinical trials without public results have not been included. Studies published in languages other than English, Italian, German, Spanish, and Portuguese have also been excluded, without specified timeframes or publish date constraints.

## Search strategy

The strategy for the database search was built for PubMed (Appendix 1) and adapted for Ovid, Cochrane Library, ISRCTN registry, and ClinicalTrials.gov. The citation list of previously published reviews and relevant works was also searched for possible papers of interest. Titles were compiled by BC and DB between the 5th of February 2025 and the 31 st of December 2025, then uploaded independently to the Rayyan platform for evaluation. Abstracts were reviewed by BC and DB, and selection was done based on the inclusion criteria. Full-text review was conducted by BC and DB, with conflicts and uncertainties being resolved via discussion between authors. Given significant gaps in our understanding of long COVID, particularly under the pediatric context, the definitions of long COVID were not subjected to scrutiny as long as the diagnosis process was performed by qualified professionals. Rather, the methodology and specificity of the biomarkers were considered, based on patients’ post-COVID inflammatory responses, to allow for the evaluation of valid future biomarker candidates.

### Data extraction and analysis

Data extracted from selected titles was compiled into a spreadsheet, built by BC. Publication information such as authors, date, and country of origin were noted. In addition, the study classification, aims, population, study group specifications, and sample size data, methodology, parameters, and outcome measures were added to the spreadsheet with the purpose of evaluating the reports of possibly relevant biomarkers (Appendix 2). Although a formal pilot test of the data extraction form was not performed, the extraction process was refined through reviewer consensus during the initial stages of data charting.

Charting was done qualitatively, and all relevant information was compiled into a table. A meta-analysis and statistical description was not the intended objective of this review, rather a standardized compilation of evidence for review. The table and diagram were designed based on the intended key findings that the authors aimed to highlight while maintaining the greatest possible consistency across variables. Diagrams were included for data illustration purposes, adding to the conventional analysis of the content derived from the collected data. A formal risk-of-bias assessment was conducted using the Modified Downs and Black Checklist [17], which is suitable for assessing methodological quality across randomized and non-randomized observational study designs. This tool was selected due to the heterogeneity of study designs included in this scoping review.

## Results

### PRISMA diagram

**Fig. 1:**
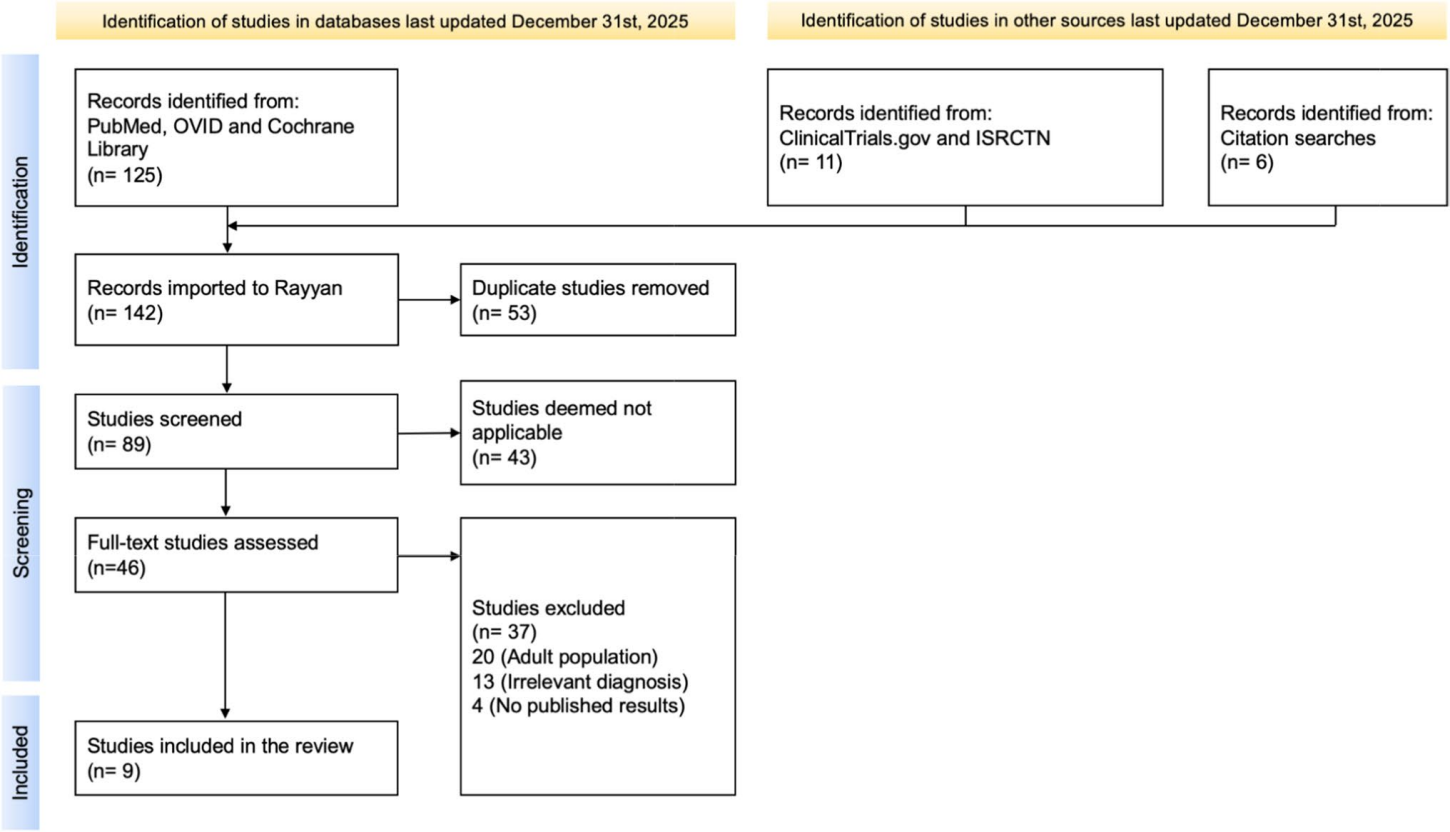
PRISMA diagram

**Fig. 2:**
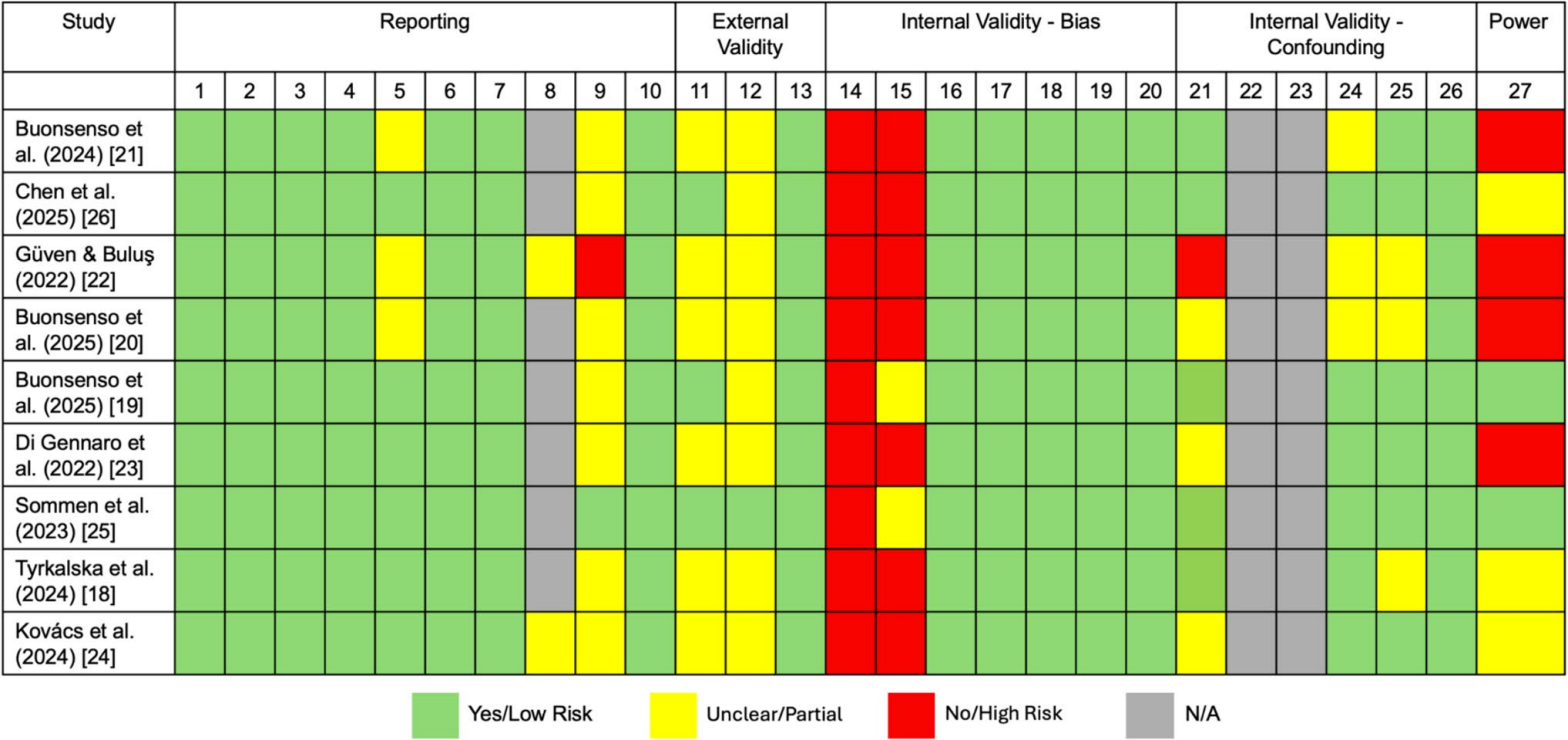
Modified Downs and Black Checklist for bias assessment (27 items across 5 domains). N/A items are not applicable to observational biomarker study designs. Itemized reference can be found in Appendix 4

In total, nine studies were deemed relevant based on the search strategy (Appendix 1) and inclusion criteria. A total of 142 articles were imported into a citation manager, wherein 53 duplicates were removed, and 46 studies were selected for full-text review post screening (Fig. 1). The eligibility criteria were met by nine papers, which were included under the individual assessment. Across the selected studies, there were 1202 patients included in the data for this review, with 664 of those being confirmed long COVID cases. The additional 538 patients were included as control and were therefore also tested for biomarkers. Detailed and complete demographic information on recruited patients was not included in every article and evaluation will be performed accordingly in this review. Risk-of-bias assessment using the Modified Downs and Black checklist indicated moderate overall methodological quality, with recurrent concerns related to external validity and confounding adjustment (Fig. 2).

A final 41 potential biomarkers were identified in this review, a few of which were reported in more than one study (Table 1). Tyrkalska et al. conducted an analysis of salivary samples from 105 children, 49 of them being classified as suffering from long COVID, and 56 healthy controls [18]. Salivary biomarkers were analyzed and nine were deemed to be significantly altered across study groups. The work of Buonsenso et al*.* emphasized the pro-inflammatory and pro-angiogenic profiles of children diagnosed with long COVID [19]. The investigation of the chemokine patterns in 34 children diagnosed with long COVID, when compared to 19 healthy controls, highlighted 11 pro-inflammatory and pro-angiogenic markers. A second title by Buonsenso et al. analyzed the cytokine profile of children suffering from long COVID symptoms [20]. The cohort recruited from the Post-COVID Outpatient Unit was composed of 10 children suffering from long COVID and 11 control children, who were either healthy or convalescent, as detailed in Table 1. In total, seven markers were found to be significantly altered between groups. Finally, Buonsenso and colleagues also identified an increase in P-selectin (CD62p) in 14 long COVID patients compared to 14 controls [21].

**Table 1:** Counts represent biomarker occurencesacross studies. Individual markers may appear more if reported by multiple studies. Extended table available in Appendix 2

Title	Inflammatory markers and pulmonary function in adolescents and young adults 6 months after mild COVID-19	Salivary biomarkers as pioneering indicators for diagnosis and severity stratification of pediatric long COVID	Distinct pro-inflammatory/pro-angiogenic signatures distinguish children with long COVID from controls	Cytokine profile in children following SARS-CoV-2 infection: Preliminary findings	Clinical and laboratory predictors of long-COVID in children: a single center retrospective study	Extended coagulation profile of children with long Covid: a prospective study	Long COVID syndrome in children: neutrophilic granulocyte dysfunction and its correlation with disease severity	Circulating Activated Platelets in Children With Long COVID: A Case-Controlled Preliminary Observation	Anti-nucleocapsid antibody levels reflect post-acute sequelae of COVID-19 symptom burden in children
Author	Sommen et al	Tyrkalska et al	Buonsenso et al	Buonsenso et al	Güven and Buluş	Di Gennaro et al	Kovács et al	Buonsenso et al	Chen et al
Year of publication	2023-01-06	2024-05-24	2025-01-24	2025-01-01	2022-01-10	2022-01-11	2024-11-27	2024-12-01	2025-10-24
Number of controls	81 (COVID-)	56 (Healthy)	19 (Healthy)	11(Healthy and convalescent)	220 (COVID+, no long COVID)	29 (recovered)	17 (COVID + convalescent)	14 (healthy)	91 (healthy)
Number of patients in disease group	367 (COVID+)	49 (long COVID)	34 (long COVID)	10 (long COVID)	31 (long COVID)	39 (long COVID at 12 weeks)	29 (long COVID)	14 (long COVID)	91 (long COVID)
Vaccination status	136 of PCC+/− 133 of PCC-	Not reported	Not reported	Not reported	Not reported	Not reported	Unspecified number	4 of LC group/12 of control group	82.61% of LC group
Age distribution	12–25 years	9–18 years	0–19 years	0–19 years	0–18 years	0–18 years	0–18 years	12–18 years	0–18 years
Reported comorbidities	Not reported	Previous psychiatric disorder (5)	Not reported	Not reported	Not reported/excluded	Non-specified comorbidities (4)	Unspecified number	Allergy (1), thyroiditis (2)	Not reported
Sample fluid	Blood	Saliva	Blood plasma	Blood	Blood	Blood	Blood	Blood	Blood plasma
List of markers significantly altered in the study group compared to the control group	MCP-1, eotaxin, IP-10 (COVID+ vs. COVID− after 6 months)	TOS, AOPP, TEACH, CUPRAC, FRAP, ADA2, tADA, alpha-amylase, total proteins	CXCL1, CXCL5, CXCL6, CXCL8, CXCL11, STAMBP1a, OSM, TNFSF11	Flt3L, CD5, uPA, CCL23, CD40, TGFα, IL-18R1	Leukocyte count, neutrophil count, monocyte count, basophil count, platelet count, and D-dimer	D-dimer	Neutrophilic granulocyte function, superoxide-producing activity, IL-6	P-selectin (CD62p)	Anti-N IgG, MIP-1α, IL-2, and IL-21

Güven and Buluş collected blood samples from 31 pediatric patients following the diagnosis of long COVID, which was compared to that obtained from 220 previously SARS-CoV-2 positive patients without long COVID symptoms [22]. The authors noted that the study group patients presented significantly increased inflammatory cell counts as well as D-dimer levels (Table 1). An additional study by Di Gennaro et al. sorted patients based on post-COVID condition (PCC) symptom persistence after 8 and 12 weeks [23]. Blood samples were analyzed for coagulation marker profiles, and patients with persistent symptoms after 12 weeks displayed significantly altered D-dimer values, in contrast to patients who had recovered. The work of Kovács et al*.* highlights the role of immune cell dysfunction on the long-term symptomology of pediatric long COVID [24]. Only 29 case group children and 17 in the convalescent control group were subjected to blood sampling, and will therefore be the only patients considered for the purposes of this review. Three markers were noted to significantly differ between children with long COVID and convalescent patients (Table 1).

Sommen and colleagues investigated the presence of inflammatory markers in children and adolescents following SARS-CoV-2 infection [25]. A total of 136 (83%) of the patients categorized as PCC positive reported having received at least one vaccine for COVID-19, compared to 133 (76%) of PCC negative patients. Findings noted no significant alteration in the tested plasma markers when PCC positive groups were compared to PCC negative patients. Nonetheless, when SARS-CoV-2 positive patients and SARS-CoV-2 negative controls were tested 6 months post-infection, significant elevations in MCP-1, eotaxin, and IP-10 were recorded (Table 1). Lastly, Chen et al*.* considered inflammatory marker variations between 91 pediatric patients diagnosed with long COVID and pre-pandemic samples of age-matched children, finding significant increases in anti-nucleocapsid IgG, MIP-1α, IL-2, and IL-21 [26].

## Discussion

In this scoping review, we found that 41 are differently expressed in children, adolescents, and young adults with long COVID compared with controls, reflecting repeated reporting of certain markers across studies. Three of the biomarkers identified in the included studies are inflammatory blood markers previously reported in samples from SARS-CoV-2 patients 6 months post-infection [27]. Utilizing biomarkers allows for critical insights into the pathology and biological mechanism of long COVID, facilitating diagnosis and guiding treatment. Elevated levels of pro-inflammatory cytokines like IL-6 and TNF-α [28] along with markers of endothelial dysfunction, such as vWF and sICAM-1 [29], have been repeatedly shown to indicate disease severity and thrombotic risk in long COVID patients. Additionally, changes in immune cell subsets [30] and markers of oxidative stress such as malondialdehyde (MDA) [31], as well as alterations in coagulation factors such as D-dimer levels [32], further contribute to our understanding of the pathophysiology of adult cases.

Nonetheless, a notable lack of clinical evidence is present for the existence of a valid biomarker associated with long COVID in younger patients specifically. Pediatric populations pose a particular challenge for medical and scientific research, yet the evidence suggesting that—contrary to previous understanding—a significant proportion of children infected with SARS-CoV-2 will develop long-term sequelae [33], stresses the need for a robust understanding of circumstantial and physiological exclusionary factors for this population. Sommen et al*.*, as explored in this review, show that despite the long-term presence of inflammatory markers in young patients following their COVID infection, the healthy controls and the study group met the criteria for post-COVID condition (PCC) to an equal percentage as denoted by the World Health Organization [34]. It becomes clear then that there is great need for a much more concrete and consistent assessment of long COVID for use by pediatric health professionals. Early analyses seem to indicate a little variation in young people compared to their adult counterparts, when it comes to both symptomology and laboratory findings; IL-6 and TNF-α, for example, have been found to be highly relevant in the long COVID inflammatory responses across age groups [35, 36]. Nonetheless, notable differences between acute phase symptomatology (pediatric cases have been shown to be clinically milder [37]), as well as immune response (a more balanced innate immune response dependent on lower ACE2 receptor expression in respiratory cells and higher natural killer cell activity having been noted in children [38, 39]), can suggest that differences in long COVID presentation will possibly be noted. Unclear clinical differences can therefore contribute to both under- and overdiagnosis of long COVID and hinder the administration of appropriate treatment plans. Potential blood and salivary markers highlighted in this review can serve as indicators of inflammatory mechanisms as well as enable early detection, risk assessment, and more personalized treatment, ultimately improving outcomes for pediatric patients.

### Inflammatory markers

**Fig. 3:**
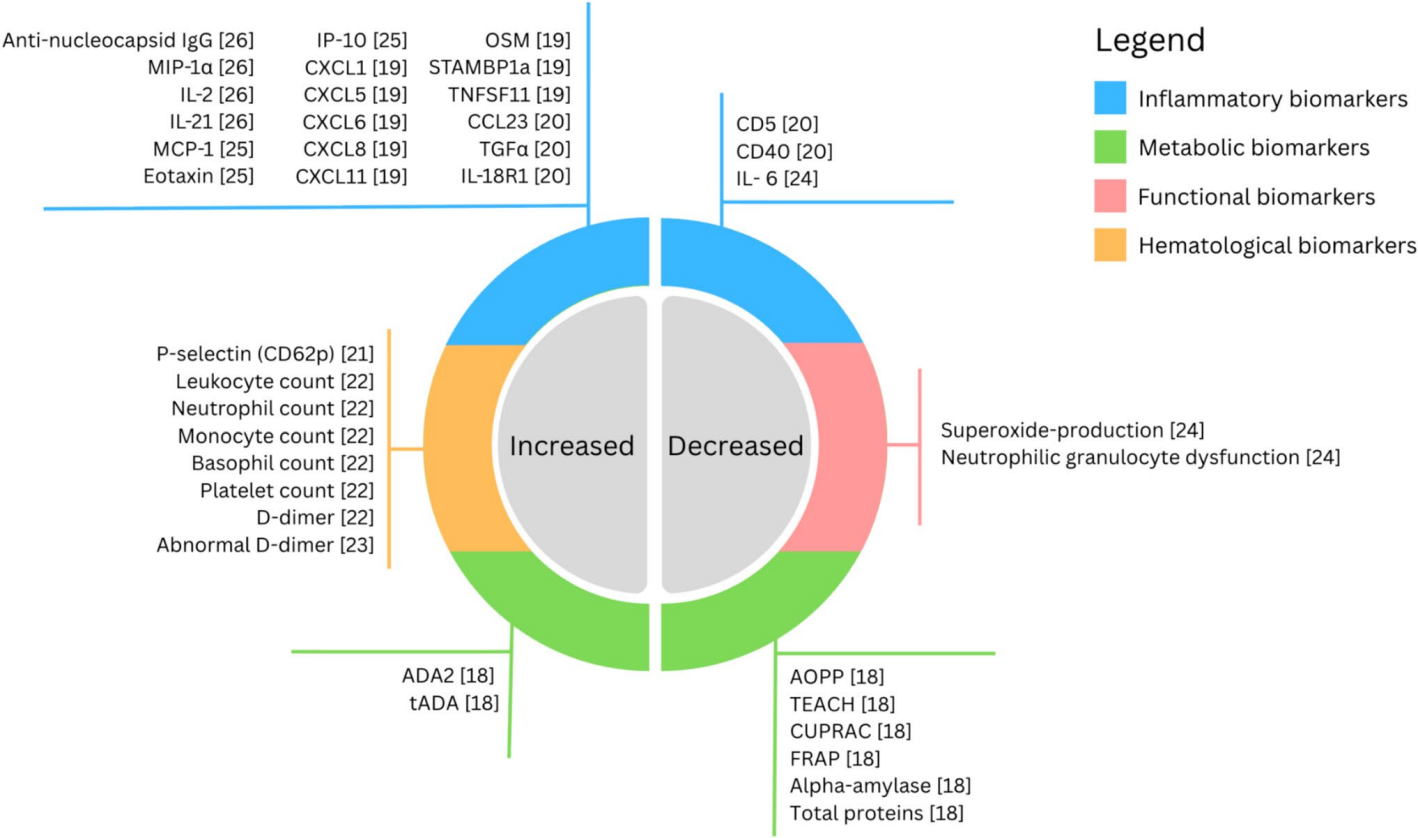
Biomarker patterns across selected studies

Pediatric cohorts across included studies show somewhat inconsistent inflammatory signatures. Buonsenso et al. show a distinct chemokine profile with elevated CXCL1, CXCL5, CXCL6, CXCL8, and CXCL11, as well as OSM, TNFSF11 (RANKL), and TGFα/STAMBP1a (Fig. 3), suggesting the presence of systemic and endothelial inflammation in the pediatric study group [19]. Neutrophil-related markers such as IL-6 and IL-8 were shown by Kovács et al. to be variably elevated [24], and a broader panel analysis conducted by Buonsenso et al. (including IL-18R1, CCL23, Flt3L, CD5, CD40) (Fig. 3) [20] supports the notion of varied, subtle immune remodeling rather than a measurable, uniform long COVID inflammatory phenotype. Similarly, limited pediatric data suggest selective immune activation, with elevated anti-nucleocapsid IgG and modest increases in MIP-1α, IL-2, and IL-21 reported in long COVID compared with healthy controls [26]. Previously conducted studies on adult patient groups seem to show rather a pattern of persistent, systemic inflammation, including persistent elevated levels of IL-6, IL-1β, TNF-α, IL-8, MCP-1, and IP-10 months after initial COVID infection [40]. The existence of larger cohort studies allows for the sorting of adult patients diagnosed with long COVID to be sorted into physiological and inflammatory phenotypes, a feat not yet achieved in pediatric groups.

Sommen et al. did not find a clear distinction in inflammatory markers between post-COVID condition (PCC)+ and PCC– patients, nor between PCC + COVID + and PCC + COVID– groups [34]. Yet, when SARS-CoV-2 positive patients and SARS-CoV-2 negative controls were tested after 6 months, elevations in MCP-1, eotaxin, and IP-10 were noted (Fig. 3). Symptom-defined PCC classification was equally frequent in the SARS-CoV-2–negative control group, bringing into question any possible biomarker diagnostic approaches. It also emphasized the background markers resulting from physiological post-infection response as well as psychosocial symptoms which can interfere with inflammatory pattern distinctions, particularly in younger patients.

#### Metabolic markers

The sample study by Tyrkalska et al. demonstrated significantly altered metabolic readouts between long COVID salivary samples and controls [18]. Study participants were found to have increased salivary tADA (as well as ADA2), which are responsible for extracellular adenosine regulation and therefore involved in pro-inflammatory response and cytokine production [41]. Chronic inflammation could also be worsened by the noted decreases in antioxidant activity measured by FRAP, CUPRAC, and TEACH, as well as increases in TOS, which suggest an imbalance in reactive oxygen species leading to oxidative stress [42]. Cellular consequences of oxidative stress range from local inflammation to autoimmune processes [43], all of which serve to confirm the suspected involvement of the immune system in pediatric long COVID, so far mostly explored in adults. The overall pattern was increased oxidant indices (e.g., TOS) with decreased antioxidant capacity (TEACH, CUPRAC, FRAP) and altered adenosine deaminase isoenzymes (tADA/ADA2 ↑; ADA1 ↔), plus lower total salivary proteins and alpha-amylase, notably changes that also correlated with symptoms of fatigue and altered school performance (Fig. 3). Previous long COVID studies have noted similar patterns in patient plasma fluid [44], but the salivary approach may allow for significantly less invasive testing, especially in the pediatric context. However, the cross-sectional study design, as well as the lack of adjustment for confounders such as infections and medications, limits the reach and establishment of causality. Localized salivary patterns may also differ from adult patterns of oxidative stress.

### Hematological markers

Güven and Buluş noted slightly higher leukocyte, neutrophil, monocyte, basophil, and platelet counts in pediatric long COVID cohorts, and in a similar manner to Di Gennaro et al., altered D-Dimer levels (Fig. 3). Although study sample sizes are small and follow-up is limited, acute-phase and post-viral inflammatory responses may, however, confound the hematologic patterns. Elevated D-dimer serves as an indicator of increased blood clotting and reflects the vascular-endothelial patterns seen clinically, particularly in long COVID patients of older age [45]. Recorded long COVID-specific increases in P-selectin (CD62p) by Buonsenso et al*.* (Table 1) indicate notable platelet activation patterns in pediatric contexts as well [46]. Yet, the hematological pattern in adult cohorts has more mechanistic anchoring, consisting of persistent thromboinflammation and presence of fibrin amyloid microclots in plasma [47, 48]. Symptom severity and endothelial implications have been more concretely established in comparison to milder, less specific pediatric shifts in counts and D-dimer, with unclear duration and uncertain specificity for long COVID.

#### Functional markers

Functional immune capacity following COVID infection has been implicated in the long-term physiological response observed in patients. Kovács et al*.* demonstrated innate immune hyporesponsiveness due to impaired neutrophilic granulocyte activity in children [24]. Specifically, long COVID-affected patients exhibited suppressed basal and PMA-stimulated superoxide-producing capacity, alongside reduced phagocytosis against *Staphylococcus aureus*. Lower superoxide production was related to greater symptom burden in the study cohort, and considering migratory function remained seemingly unaffected, cells may be affected by intracellular activation rather than chemotactic recruitment. Overall, Kovács et al*.* suggest functional dampening may be reflecting altered immune post-COVID response, rather than active acute-phase inflammation. This stands in contrast to adult cohort studies which have demonstrated rather hyperactive neutrophils, exaggerated superoxide and NET formation, leading to endothelial injury and microthrombosis reports [49, 50]. This serves to emphasize the possibility of inherent physiological differences in age-specific long COVID manifestations and subsequent symptomological manifestations. Larger, longer-term studies would allow for a more concrete picture of functional implications and pediatric-specific response.

#### Critical assessment

The studies in this review provide imperative initial insight into the physiological patterns behind pediatric long COVID; they are limited by small sample sizes and heterogeneous designs. Most cohorts enrolled less than 100 participants from single centers, lacking statistical power or pre-specified hypotheses. This severely limits the reproducibility of the results. The larger case study by Sommen et al*.* enrolled several hundred patients but introduced through its population-wide approach the matter of high heterogeneity that may be behind the almost equal prevalence of post-COVID condition (PCC) in infected and uninfected participants [34]. Conversely, the other studies, such as Buonsenso et al*.* [19], recruited from specialized clinics which could introduce a selection bias that fails to reflect community phenotypes of pediatric long COVID. As demonstrated in Fig. 2, though most studies demonstrated adequate reporting and biomarker measurement methods, the risk-of-bias patterns point to ongoing challenges in study design, including limited representativeness and incomplete adjustment for confounders. These limitations should be considered when interpreting the current biomarker evidence.

In the case of the salivary analysis conducted by Tyrkalska and colleagues, a bias in analytics could have emerged from the use of stepwise regression and ROC modeling without the addition of a longitudinal follow-up, as it limits the establishment of causality [18]. Confounding variables present an additional limitation, since few titles controlled for factors such as acute COVID-19 severity, vaccination status, comorbidities, age, or pubertal stage. Reference ranges for biomarker studies remain poorly established in the literature and limit compatibility. In unison, the presence of the mentioned limitations highlights the exploratory, initial stage of pediatric long COVID biomarker research and the need for further studies on a larger scale.

### Limitations

Natural limitations of the study result from its scoping nature, providing an overlook at the available studies but lacking the detailed statistical evaluation of a meta-analysis. In addition, the included studies use heterogeneous comparator groups (healthy controls, SARS-CoV-2–negative controls, convalescent COVID-19 patients, pre-pandemic samples). This heterogeneity limits direct comparability of biomarker findings across studies. Therefore, this protocol may not be appropriate for certain types of research questions. Nonetheless, this study fulfills the purpose of creating a basis of knowledge reference for future studies by highlighting gaps in the literature that can guide future works and improve completeness of the field.

## Conclusion

This review suggests the presence of objective biological phenomena manifested in children, adolescents, and young adults with long COVID, demonstrated across a variety of studies and patient populations. The significance of physiologically measurable markers in the identification and characterization of long COVID suggests that SARS-CoV-2 has direct implications on immune system function even months post-infection. This review can serve as a basis to guide future diagnostic biomarkers and see how they evolve longitudinally after the infections, with or without treatments, and serve as possible correlates of improvement in future pharmacological trials.

## Supplementary Information

Below is the link to the electronic supplementary material.

ESM 1 (DOCX 2.61 MB)

ESM 2 (XLSX 2.70 MB)

## Data Availability

No datasets were generated or analysed during the current study.

## Abbreviations

ADA2: Adenosine deaminase 2
AOPP: Advanced oxidation protein products
CUPRAC: Cupric reducing antioxidant capacity
FRAP: Ferric reducing ability of plasma
GI: Gastrointestinal
MDA: Malondialdehyde
ME/CFS: Myalgic encephalomyelitis/chronic fatigue syndrome
PASC: Post-acute sequelae of SARS-CoV-2 infection
PCC: Post-COVID condition
PCR: Polymerase chain reaction
POTS: Postural orthostatic tachycardia syndrome
PRISMA-ScR: Preferred Reporting Items for Systematic reviews and Meta-Analyses extension for Scoping Review
RANKL RANK: ligand
RT-PCR Reverse: transcription polymerase chain reaction
tADA: Total adenosine deaminase
TEACH: Trolox equivalent antioxidant capacity in the hydrophilic fraction
TGFα: Transforming growth factor alpha
TOS: Total oxidant status
uPA: Urokinase-type plasminogen activator
WHO: World Health Organization
