# Supplementary material for: Biomarkers of long COVID in children and young adults: a scoping review
Source: Eur J Pediatr. 2026 Feb 16;185(3):132. doi: 10.1007/s00431-026-06789-7 (PMC12909393; doi:10.1007/s00431-026-06789-7)
Supplement: Supplementary file 1 — (DOCX 2.61 MB) [file 431_2026_6789_MOESM1_ESM.docx]

**Appendix 1**

PubMed search strategy*. Additional strings used in other databases included in Appendix 3.

| **Number** | **Search Terms** | **Hits** |
| --- | --- | --- |
| #1 | "post-acute covid-19 syndrome"[MeSH Terms] OR "long covid"[Text Word] | 9,472 |
| #2 | ("post-acute covid-19 syndrome"[MeSH Terms] OR "long covid"[Text Word]) AND "humans"[All Fields] | 6,429 |
| #3 | "Biomarkers"[MeSH Terms] AND ("Post-Acute COVID-19 Syndrome"[MeSH Terms] OR "long covid"[Text Word]) | 239 |
| #4 | "Biomarkers"[MeSH Terms] AND ("post acute covid 19 syndrome"[MeSH Terms] OR "long covid"[Text Word] OR "post acute covid 19 syndrome"[MeSH Major Topic]) | 239 |
| #5 | (("Biomarkers"[MeSH]) AND  ("Post-Acute COVID-19 Syndrome"[MeSH] OR "long covid"[Text Word] OR "chronic COVID"[Text Word]  OR "post COVID"[Text Word] OR "Post-Acute SARS-COV-2 Syndrome"[Text Word]  OR "post SARS"[Text Word] OR "long term SARS"[Text Word]  OR "long term COVID"[Text Word] OR "prolonged SARS"[Text Word]  OR "prolonged COVID"[Text Word])) | 365 |
| **#6** | **(("Biomarkers"[MeSH]) AND**  **("Post-Acute COVID-19 Syndrome"[MeSH] OR "long covid"[Text Word] OR "chronic COVID"[Text Word]**  **OR "post COVID"[Text Word] OR "Post-Acute SARS-COV-2 Syndrome"[Text Word]**  **OR "post SARS"[Text Word] OR "long term SARS"[Text Word]**  **OR "long term COVID"[Text Word] OR "prolonged SARS"[Text Word]**  **OR "prolonged COVID"[Text Word])) AND**  **(("pediatr*"[All Fields]) OR ("paediatr*"[All Fields])**  **OR ("child*"[All Fields]) OR ("infant*"[All Fields])**  **OR ("toddler*"[All Fields]) OR ("adolescen*"[All Fields])**  **OR ("youth*"[All Fields]) OR ("teen*"[All Fields]))** | **69** |

*****Language and date filters were not utilized. Titles written in languages other than English, Italian, Spanish, Portuguese and German were excluded during screening.

**Appendix 2**

**Original Spreadsheet included in attached files**

**Appendix 3**

| **Cochrane string:** | **Biomarkers AND ("Post-Acute COVID-19 Syndrome" OR "long covid" OR "chronic COVID" OR "post COVID" OR "Post-Acute SARS-COV-2 Syndrome" OR "post SARS" OR "long term SARS" OR "long term COVID" OR "prolonged SARS" OR "prolonged COVID") AND (pediatric OR paediatric OR child OR infant OR toddler OR adolescent OR youth OR teen)** |
| --- | --- |
| **OVID string:** | **1. exp Biomarkers/**  **2. exp "Post-Acute COVID-19 Syndrome"/ OR "long covid".mp.**  **3. "chronic COVID".mp. OR "post COVID".mp. OR "Post-Acute SARS-COV-2 Syndrome".mp. OR "post SARS".mp. OR "long term SARS".mp. OR "long term COVID".mp. OR "prolonged SARS".mp. OR "prolonged COVID".mp.**  **4. 2 OR 3**  **5. exp Pediatrics/ OR pediatr*.mp. OR paediatr*.mp. OR child*.mp. OR infant*.mp. OR toddler*.mp. OR adolescen*.mp. OR youth*.mp. OR teen*.mp.**  **6. 1 AND 4 AND 5** |
| **ClinicalTrials.gov string:** | **Biomarkers AND ("Post-Acute COVID-19 Syndrome" OR "long covid" OR "chronic COVID" OR "post COVID" OR "Post-Acute SARS-COV-2 Syndrome" OR "post SARS" OR "long term SARS" OR "long term COVID" OR "prolonged SARS" OR "prolonged COVID") AND (pediatr* OR paediatr* OR child* OR infant* OR toddler* OR adolescen* OR youth* OR teen*)** |
| **ISRCTN string:** | **Biomarkers AND ("Post-Acute COVID-19 Syndrome" OR "long covid" OR "chronic COVID"**  **OR "post COVID" OR "Post-Acute SARS-COV-2 Syndrome" OR "post SARS"**  **OR "long term SARS" OR "long term COVID" OR "prolonged SARS" OR "prolonged COVID")**  **AND (pediatr* OR paediatr* OR child* OR infant* OR toddler***  **OR adolescen* OR youth* OR teen*)** |

**Appendix 4**

**Item Reference Guide for Traffic Light Bias-Assassment Plot. Assessment was conducted in accordance with the modified Downs and Black checklist.**

| **#** | **Domain** | **Item Description** |
| --- | --- | --- |
| **1** | **Reporting** | **Aim/hypothesis clearly stated** |
| **2** | **Reporting** | **Main outcomes clearly described** |
| **3** | **Reporting** | **Patient characteristics clearly described** |
| **4** | **Reporting** | **Interventions/exposures clearly described** |
| **5** | **Reporting** | **Principal confounders stated** |
| **6** | **Reporting** | **Main findings clearly described** |
| **7** | **Reporting** | **Estimates of random variability** |
| **8** | **Reporting** | **Adverse events reported** |
| **9** | **Reporting** | **Characteristics of lost patients** |
| **10** | **Reporting** | **Probability values reported** |
| **11** | **External Validity** | **Subjects representative** |
| **12** | **External Validity** | **Subjects prepared to participate representative** |
| **13** | **External Validity** | **Staff/facilities representative** |
| **14** | **Internal Validity - Bias** | **Blinding of subjects** |
| **15** | **Internal Validity - Bias** | **Blinding of outcome assessors** |
| **16** | **Internal Validity - Bias** | **Data dredging addressed** |
| **17** | **Internal Validity - Bias** | **Appropriate statistical tests** |
| **18** | **Internal Validity - Bias** | **Compliance reliable** |
| **19** | **Internal Validity - Bias** | **Main outcome measures valid/reliable** |
| **20** | **Internal Validity - Bias** | **Cases and controls from same population** |
| **21** | **Internal Validity - Confounding** | **Recruitment over same period** |
| **22** | **Internal Validity - Confounding** | **Randomization adequate** |
| **23** | **Internal Validity - Confounding** | **Allocation concealment** |
| **24** | **Internal Validity - Confounding** | **Adjustment for confounding** |
| **25** | **Internal Validity - Confounding** | **Losses to follow-up accounted** |
| **26** | **Internal Validity - Confounding** | **Sufficient follow-up period** |
| **27** | **Power** | **Sufficient sample size/power** |
